# Effect of Arginase-1 Inhibition on the Incidence of Autoimmune Diabetes in NOD Mice

**Published:** 2018-08-01

**Authors:** Luis F Hernandez, Peter Buchwald, Midhat H Abdulreda

**Affiliations:** 1 Diabetes Research Institute, University of Miami Miller School of Medicine, USA; 2 Department of Molecular & Cellular Pharmacology, University of Miami Miller School of Medicine, USA; 3 Department of Surgery, University of Miami Miller School of Medicine, USA

**Keywords:** nor-NOHA, T1D, NOD, Incidence rate, Arginine, Metabolism, Ornithine, Nitric oxide, Inflammation, Macrophages

## Abstract

Metabolism of the amino acid L-arginine is implicated in many physiological and pathophysiological processes including autoimmune conditions such as type 1 diabetes (T1D). Alternate arginine metabolism through the citrulline-nitric oxide (NO) or the ornithine pathways can lead to proinflammatory or immune regulatory effects, respectively. In this report, we blocked the arginine-ornithine metabolic pathway by inhibiting the enzyme arginase-1 with Nω-hydroxy-nor-arginine (nor-NOHA) to make arginine more available to the alternate citrulline pathway for augmented NO production and increased incidence of autoimmune T1D in female non-obese diabetic (NOD) mice. Unexpectedly, mice receiving nor-NOHA did not develop diabetes although increased NO production is proinflammatory and expected to increase diabetes incidence. These results warrant further studies of the mechanism of action of nor-NOHA, and highlight its potential as a therapeutic agent for the treatment or prevention of T1D.

## Introduction

**Figure 1:**
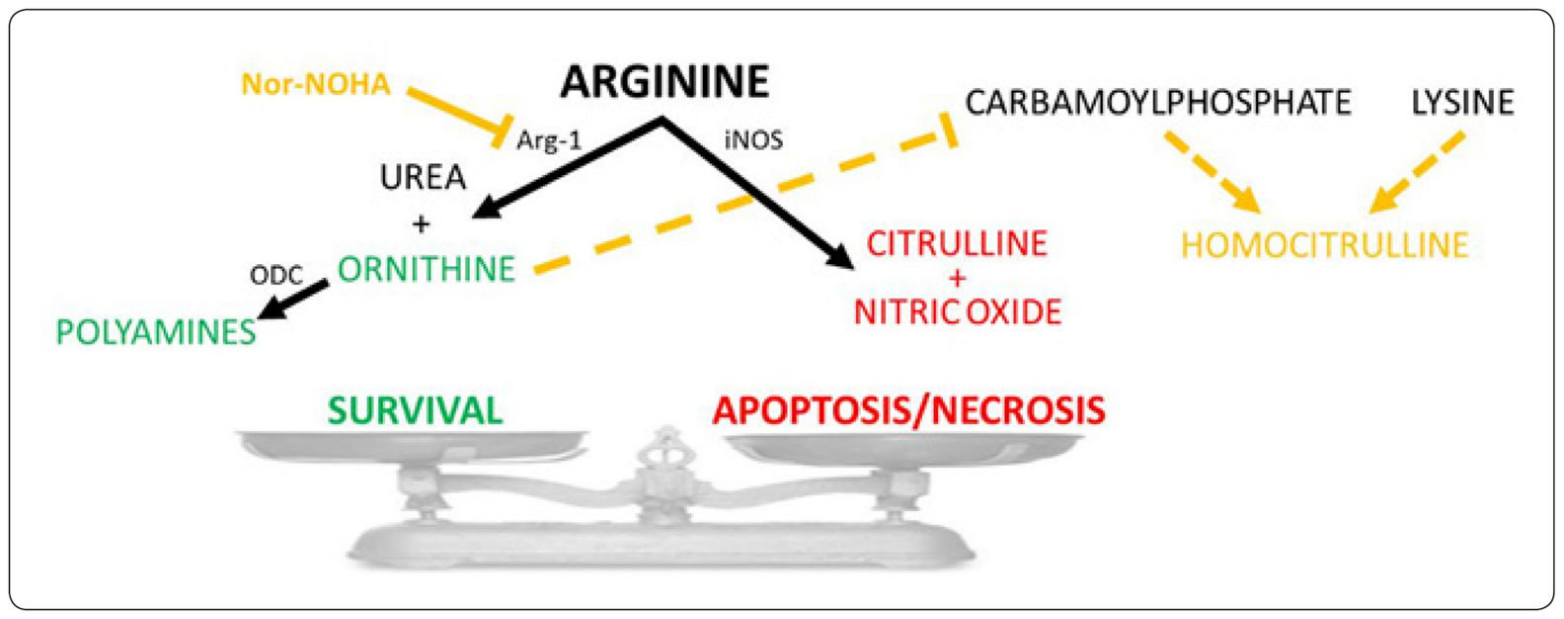
Arginine metabolism through the urea-ornithine and citrulline-NO pathways and its consequences on diabetes incidence in NOD mice. Simplified scheme/model of alternate arginine metabolism either through the urea-ornithine or the citrulline-nitric oxide (NO) pathways leading to anti-or pro-inflammatory effects, respectively, and associated decrease or increase in diabetes incidence. The scheme also illustrates the effect of nor-NOHA on arginase-1 (Arg-1) and the production of ornithine and downstream polyamines by ornithinedecarboxylase (ODC). The citrulline-NO pathway is catalyzed by induced nitric oxide synthase (iNOS). Citrulline is also produced from carbamoylphosphate and ornithine. When ornithine is reduced through Arg-1 inhibition by nor-NOHA, homocitrulline is produced from carbamoylphosphate and lysine.

The role of L-arginine metabolism in autoimmune diseases has recently become of interest. Specifically, metabolic products of two alternate arginine metabolism pathways (Figure 1) are implicated in both proinflammatory/autoimmune and immune regulatory/tolerogenic immune responses. The arginine-citrulline-nitric oxide (NO) pathway favors proinflammatory polarization of innate (M1-like) and adaptive (Th1/Th17) immune cells. For example, when enough arginine substrate is available and the enzyme NO synthase (NOS or iNOS) is upregulated, NO production increases in pro-inflammatory M1 macrophages. Depending on its level, NO can be either pro- or anti-inflammatory. Whereas, high levels of NO are proinflammatory and cytotoxic, and promote both apoptosis and necrosis in target cells [1,2], low levels have been shown to have anti-inflammatory effects [3–5]. On the other hand, the alternate ornithine pathway of arginine metabolism favors polarization towards immunoregulatory function in immune cells such as regulatory T cells (Tregs) and M2 macrophages, which are essential during resolution of inflammation, tissue healing, and immune tolerance induction/maintenance. When the enzyme arginase-1 is overexpressed, arginine is metabolized to urea, ornithine, and downstream polyamines that are crucial building blocks during cell proliferation and maintenance of tissue homeostasis. Therefore, we hypothesized that administration of an arginase-1 inhibitor should block the urea-ornithine pathway making arginine more available for the alternate citrulline-NO pathway for increased production of NO and associated tissue inflammation and, ultimately, increased incidence of autoimmune diabetes (Figure 1).

Nor-omega-hydroxide-L-arginine (nor-NOHA) is a competitive inhibitor of arginase-1 that should increase arginine availability to the NO pathway, whereby, increasing the production of NO [6]. To our knowledge there are no available reports on the effect of nor-NOHA on the autoimmune process in animal models of type 1 diabetes (T1D). The non-obese-diabetic (NOD) mouse is a widely used model of human T1D and provides an attractive opportunity to investigate the effect of nor-NOHA on the incidence of autoimmune diabetes [7,8]. The diagram shown in Figure 1 illustrates a working model of the effect arginine metabolism and nor-NOHA treatment on diabetes incidence in diabetes-prone female NOD mice. In the current study, we investigated arginine metabolism and the spontaneous incidence of autoimmune diabetes as a function of age in female NOD mice, and evaluated the effect of nor-NOHA treatment on diabetes incidence.

## Materials and Methods

### Animals and treatments

Female NOD mice of around 8 weeks of age were purchased from Taconic Biosciences (USA) and Jackson Laboratories (USA) and housed in groups of five with access to food and water *ad libitum.* The room temperature was kept at 23°Celsius and the light-dark cycle was 12/12 hours. All animal procedures were approved by the IACUC of the University of Miami. Nor-NOHA was obtained from Cayman Chemicals (USA). It was dissolved in PBS (phosphate buffered saline), and injected intraperitoneally at the dose of 30mg/kg every other day until 12 doses were completed. Blood sugar measurements were obtained using Contour Next® glucometer and strips (Bayer). A drop of blood was obtained by pricking the distal end of the tail with a 25G hypodermic needle and the drop was applied to the blood glucose strip. Non-fasting blood sugar levels were measured every two days before and during the treatment period (with nor-NOHA or PBS), and then once in the week after, and once 172 days after the beginning of the treatment. Mice were considered diabetic after 3 consecutive readings of hyperglycemia (>300mg/dL) [7].

### Metabolomics analysis

Blood concentration data for arginine, its derivatives, and other metabolites used here were obtained in an untargeted metabolomics study by LC–MS as described previously in detail [9]. In brief, repeated blood samples were collected prospectively from NOD mice at 6, 11, 16, 21, and 26 weeks of age. Blood glucose was also measured at least twice weekly to monitor diabetes incidence in the same mice. Data from blood samples obtained in mice that developed diabetes by 26 weeks of age were pooled into the diabetic (progressor) group, and those from mice remained normoglycemic by the same age were considered as non-diabetic controls (non-progressors) in the analysis. We did not conduct analysis in mice older than 26 weeks of age to avoid potential confounding issues related to long-standing diabetes and the insulin therapy in the diabetic mice. The metabolomics data in the diabetic mice (up to 26 weeks of age) were expressed as fold increase relative to non-diabetic controls. See reference [9] for complete details.

### Statistical analysis

Data were plotted and analyzed using Prism 6 version 6.07 (GraphPad Software, Inc; La Jolla California USA). Kaplan-Meyer survival curves were used to calculate the median age (in weeks) of spontaneous onset of diabetes in the total pooled populations of female NOD mice obtained from the different vendors. Additional analysis of the median age of diabetes onset was also performed based on the cumulative percent of diabetic mice over time assuming a Hill-type sigmoidal dependence. Estimations of the incidence rate of diabetes was done with onset data binned per week and obtained using the slope values of linear regression analysis of the cumulative percent of diabetic mice in two different regimes using a rate-change point identified by a linearized biexponential model as previously described in detail [10,11]. Segmental linear regression analysis was carried out using the minimum sum of squared errors method to identify the change in inflection of the data (i.e., transition points between the linear regimes). The diabetes incidence rate or probability in each regime (i.e., the estimated number of mice developing diabetes during a specified age-range) was calculated based on the slope value multiplied by the age-range (in weeks) and divided by the total number of mice remaining normoglycemic at the beginning of the chosen age range. Comparisons of the slopes of the linear regressions was done by F-test. Pair-wise comparisons of the treatment groups were by Student t-test or ANOVA followed by post hoc tests (e.g., Holm-Sidak method). Data are presented as means±SEM. Asterisks indicate significant difference at p <0.05 level.

## Results

### Changes in arginine metabolism in diabetic progressor vs. non-progressor female NOD mice

**Figure 2:**
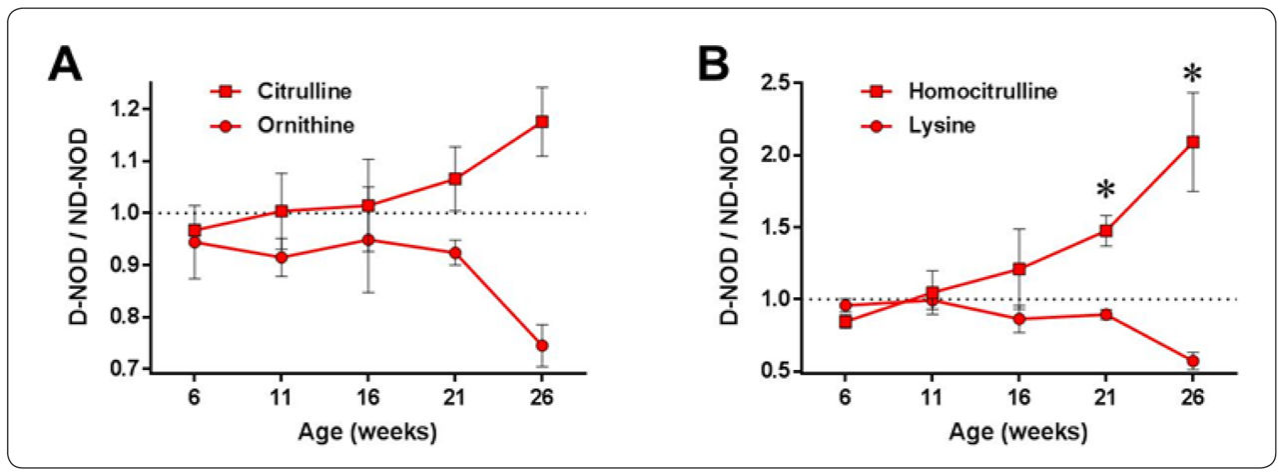
Longitudinal changes in arginine metabolites and related amino acids in female NOD mice. Ratios of (A) ornithine and citrulline and (B) lysine and homocitrulline levels (measured by LC–MS) in the blood of diabetic (D-NOD) female NOD mice relative to those in non-diabetic (ND-NOD) control counterparts at 6, 11, 16, 21, and 26 weeks of age. Data presented as means±SEM (n=3 ND-NOD and 4 D-NOD mice).

We assessed the metabolism of arginine and other amino acids as part of an untargeted longitudinal metabolomics study [9] in repeated blood samples obtained from female NOD mice that did or did not progress to autoimmune diabetes during a follow up period between 6 and 26 weeks of age. The overall results showed relatively similar metabolic profiles between the groups before onset of diabetes (i.e., between 6 and 16 weeks) (Figure 2). However, differences in arginine metabolites and other amino acids became evident between 21 and 26 weeks coincident with diabetes onset. At week 26, ornithine was reduced by ~30% and citrulline was elevated by ~20% in diabetic mice (D-NOD) compared to non-diabetic controls (ND-NOD) (Figure 2A). Lysine was also reduced by ~50% while homocitrulline was significantly increased at weeks 21 (50%) and 26 (110%) in diabetic mice compared to non-diabetic counterparts (Figure 2B).

### Incidence of autoimmune diabetes in NOD mouse population as a function of age

**Figure 3:**
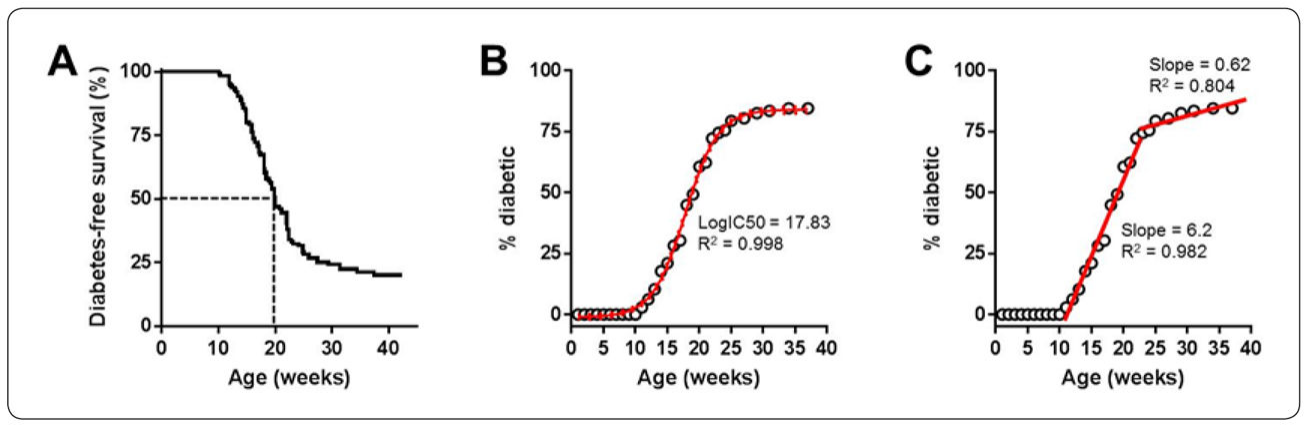
Incidence of autoimmune diabetes with age in NOD mouse population. (A) Kaplan-Meyer survival curves showing the time-profile of diabetes occurrence in a total pooled population of NOD mice (n=126) monitored up to 43 weeks of age (last occurrence of diabetes was at 37 weeks). Dotted lines represent the median age of diabetes-free survival (alternatively, median age of diabetes onset) in the total population. (B) Diabetes incidence in the NOD mice (shown in A) expressed as cumulative percent (binned per week; open circles) of the total population. The data were fit with a four-parameter sigmoid function (red solid line with 95% confidence levels shown as dots). (C) Same data as in B with best fit multi-segment linear fit (red lines) highlighting two distinct regimes (between the ages of 10 to 23 and 24 to 43 weeks) with different diabetes incidence rates. R2 values are included to show the goodness of fit of the linear and nonlinear regression analyses.

We tracked the spontaneous incidence of autoimmune diabetes in a total pooled population of young female NOD mice (n=126) starting around 8 weeks of age. Mice that received no treatment started to develop diabetes around 10 weeks of age. By 38 weeks, approximately 80% of the mice became diabetic and the remaining 20% maintained normoglycemia up to ~43 weeks of age (Figure 3A). According to Kaplan-Meyer survival curve analysis, in this cohort the median age of diabetes onset was 19.9 weeks in the total population of female NOD mice aged up to 43 weeks. Further analysis of diabetes incidence based on the cumulative percentage of diabetic mice during the same age range using nonlinear regression analysis (fit with a Hill curve) showed a median age of diabetes onset of 17.8 weeks (Figure 3B). While these analyses yielded estimates of the age of diabetes onset in the total population between 8 and 43 weeks of age, they could not provide estimates of the likelihood of diabetes development in the remaining ~20% fraction of mice that maintained normoglycemia up to this age. Data indicate that, at least in these NODs, the rate of diabetes onset is not completely flattened out (i.e., zero), and there is a decreasing, but non-zero chance of still developing diabetes in older female NOD mice. Therefore, we adopted a multi-linear model to fit the data along two distinct linear regimes from 10 to 23 and 24 to 43 weeks of age with a rate-of-change point selected as to give the best fit of the data (Figure 3C) also see Methods. The slopes of the first and second regimes obtained this way (6.2 and 0.62, respectively) were significantly different (p<0.00001), which indicated varying diabetes incidence rates before and after 23 weeks of age in this pooled NOD mouse population.

### The effect of nor-NOHA on the incidence of autoimmune diabetes in old NOD mice

**Figure 4:**
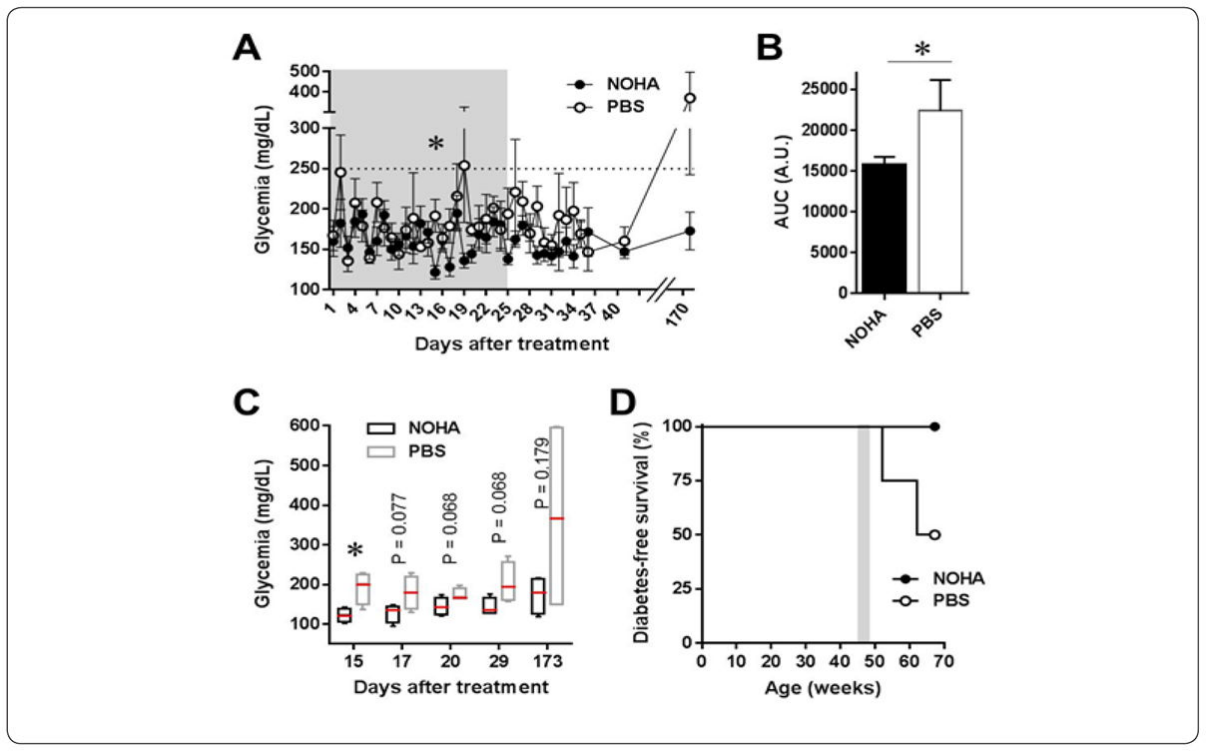
The effect of nor-NOHA on glycemia and diabetes incidence in old non-diabetic female NOD mice. (A) Non-fasting blood glucose levels during a treatment period (gray area) with nor-NOHA (NOHA; filled black symbols) or PBS control (open symbols) in ~45 weeks old female NOD mice (n =4 mice each). (B) Area under the curve (AUC) values of data shown in A. Asterisks denote significant difference with p < 0.05 by Student t-test in A and by F-test to compare variances in B (F =18.45 (3/3)). (C) Non-fasting glycemia in nor-NOHA and PBS-treated mice at selected time-points during and after treatment (shown as Box and Whiskers plot with red lines indicating the median). (D) Kaplan-Meyer survival curves showing diabetes-free survival (i.e., remaining normoglycemic) in these mice during and after treatment (gray area) with nor-NOHA or PBS. The median age of diabetes onset was 64.7 weeks in PBS-treated mice and remained undefined in the nor-NOHA group.

Normoglycemic old female NOD mice were treated with nor-NOHA (30mg/kg, i.p.) or PBS as control every other day for 24 days starting around 45 weeks of age. During the treatment period, the non-fasting blood glucose levels in both treatment groups fluctuated but remained within “normal” glycemia range (<300mg/dL). During a 6-week follow up period, the mean non-fasting glycemia (measured every 2 days) in the PBS-treated mice was higher on 8 occasions and lower on 7 occasions than in the nor-NOHA group (Figure 4A). While all mice remained normoglycemic during and after the treatment period, there was a tendency toward lower mean non-fasting glycemia in the nor-NOHA-treated mice compared to the PBS controls as indicated, for example, by the blood glucose AUC data (Figure 4B) and the median values of non-fasting blood glucose at selected time-points with marginally significant difference (Figure 4C). Moreover, further follow up of the mice up to 25 weeks after treatment initiation showed that 50% of the PBS-treated mice became diabetic, while 100% of the nor-NOHA-treated mice remained normoglycemic (Figure 4D).

## Discussion

Female NOD mice are used extensively as a model for human autoimmune T1D [12–17]. Numerous studies have shown that under specific-pathogen-free conditions typically 70–90% of female NOD mice develop diabetes spontaneously by 45 weeks of age [9,11]. Consistent with this, we found an 80% diabetes incidence rate in a population of 126 female NOD mice aged up to 43 weeks (Figure 1). While most studies are conducted in NOD mice that develop diabetes before this advanced age, the remaining 10–30% of NOD mice that maintain normoglycemia by age 45 weeks are typically discarded from studies because of their reduced incidence of diabetes. Histological analysis of the pancreas from these older non-diabetic female NOD mice have shown evident mononuclear cell infiltrate around relatively intact islets [11]. This observation, also known as peri-insulitis, raises the possibility for local immune regulatory processes that may slow down the autoimmune attack against the insulin-producing beta cells, leading to reduced diabetes incidence. It is unclear however what specific diabetes incidence rate old female NOD mice have because long-term follow up beyond 45 weeks of age is rarely done, likely due to experimental and other logistical considerations. We reasoned that old non-diabetic female NOD mice provide a useful model to investigate the effects of different interventions aimed at changing the incidence of autoimmune diabetes beyond the age range where the probability of diabetes onset is significantly higher due to more aggressive autoimmune responses against the beta cells. Studies in older non-diabetic NOD mice may also be more representative of immune processes in adults, and may be particularly pertinent to the investigation of immune regulatory function and immune tolerance.

Arginine metabolism has been implicated in both proinflammatory and immune regulatory processes. Consequently, alternate arginine metabolism through the ornithine or citrulline pathways may lead, respectively, to decreased or increased incidence of autoimmune diabetes (Figure 1). Proinflammatory Th1/Th17 cytokines activate iNOS which produces nitric oxide (NO) from L-arginine through the citrulline-NO metabolic pathway in proinflammatory immune cells. NO-producing immune cells such as M1 macrophages can damage the pancreatic islets during diabetes development (McDaniel et al., 1996). Citrulline is also produced from carbamoylphosphate and ornithine; however, when ornithine is less abundant homocitrulline is produced from carbamoylphosphate and lysine. While no significant variations of citrulline and ornithine levels in the blood have been reported in association with aging [18], homocitrulline has been shown to increase progressively with age and has been suggested as a biomarker of aging-associated inflammation [19,20]. Homocitrulline has also been implicated in the pathogenesis of autoimmune arthritis [21,22], but little is known about its involvement in T1D. Our current findings, however, showing significantly elevated homocitrulline levels in diabetic NOD mice (Figure 2B) suggest its possible involvement, in addition to arginine metabolism, in the inflammation during the pathogenesis of T1D, and this involvement is likely to have persisted in the mice aged beyond 26 weeks old. Alternatively, Th2 cytokines induce arginase-1, which hydrolyses arginine into urea and ornithine, and in turn drives immune cell polarization towards regulatory function. Ornithine is also a precursor for polyamines that are required for cell proliferation and maintenance (Classen et al., 2009). Consistently, our current findings showed increased levels of citrulline and reduced levels of ornithine in diabetic female NOD mice compared to non-diabetic counterparts (Figure 2A). Therefore, we investigated whether reducing ornithine through arginase-1 inhibition by nor-NOHA treatment and promoting the alternate arginine-citrulline-nitric oxide (NO) pathway will influence the incidence of autoimmune diabetes in >45 weeks old female NOD mice.

Based on our analysis of the incidence of diabetes in female NOD mouse population, we estimated incidence rates of approximately 64% and 43% in mice aged 10 to 23 and 24 to 43 weeks, respectively. These predicted rates were consistent with those observed in the total NOD population for each of the age ranges (66% and 31%; Figure 3A). Using the same approach and by extrapolating the data in the second linear regime up to 68 weeks of age, we estimated that ~50% of the NOD mice remaining normoglycemic by age 43 weeks (i.e., 45 out of 126) will become diabetic by 68 weeks. Consistent with this predicted rate, our experimental data showed that half of the control PBS-treated mice became diabetic by 68 weeks of age (Figure 4D); whereas, unexpectedly, all the nor-NOHA-treated mice remained normoglycemic during the same follow up period. This was surprising because blocking the arginine-ornithine pathway makes arginine more available for the alternate arginine-citrulline-NO pathway, and was expected to increase NO production and associated pro-inflammatory effects causing increased diabetes incidence. Contrary to this, our current findings suggested that nor-NOHA treatment seemingly protected the mice from autoimmune diabetes. While more studies are needed to further explain these findings, and while we did not measure citrulline and other arginine metabolites and polyamines in the >45 weeks old mice, the current results are consistent with other studies using nor-NOHA in animal models of inflammatory conditions other than T1D. For instance, nor-NOHA was reported to ameliorate inflammation in (i) the adipose tissues of mice on a high-fat-diet [23], (ii) the bronchi in an animal model of bronchial asthma [24], and (iii) the arteries of another model of arthritis [25]. Therefore, while we speculate that arginase-1 inhibition in our study likely resulted in increased production of NO, the relatively short nor-NOHA treatment regimen we used may have resulted in moderate increase in NO levels, which has been shown to have anti-inflammatory rather than proinflammatory effects [3–5]. Moreover, nor-NOHA could have interfered with the production of the proinflammatory eukaryotic translation initiation factor 5A (eIF5A) to prevent the autoimmune response, as blocking eIF5a with siRNA was recently shown to reduce mouse mortality by LPS [26], and its knockdown increased resistance to streptozotocin-induced diabetes in mice [27]. Moreover, treatment with di-fluoromethyl-ornithine (DFMO; a blocker of ornithine decarboxylase which transforms ornithine into putrescine/hypusine) or with N1-guanyl-1,7-diaminoheptane (an inhibitor of the enzyme desoxyhypusine which is critical for the activation of eIF5A) was also shown to reduce insulitis and the incidence of autoimmune diabetes in young NOD mice [28,29]. Interestingly, the eIF5A inhibitor DFMO is currently in clinical trials to evaluate its effect on halting the progression of autoimmune diabetes in children with new onset T1D (Clinical Trials.gov; study identifier NCT02384889).

## Conclusion

In summary, our current study showed that arginine metabolism is altered in diabetes-prone NOD mice; the arginine-citrulline-NO pathway was upregulated, whereas the alternate arginine-ornithine pathway was downregulated in diabetic female NOD mice as compared to their non-diabetic counterparts. A 24-day treatment with the arginase-1 inhibitor nor-NOHA aimed to further upregulate the citrulline-NO pathway unexpectedly reduced the incidence of autoimmune diabetes in older diabetes-prone female NOD mice. These findings warrant further investigation of the mechanism of action of nor-NOHA on the autoimmune process in diabetes, and highlight its potential as a therapeutic agent for the treatment or prevention of T1D.

## Abbreviations

nor-NOHA: Nω-hydroxy-nor-arginine
T1D: Type 1 Diabetes
NOD: Non-Obese-Diabetic
D-NOD: Diabetic NOD
ND-NOD: Non-Diabetic NOD
NO: Nitric Oxide
iNOS: Induced Nitric Oxide Synthase
Arg-1: Arginase-1
ODC: Ornithinedecarboxylase
DFMO: Di-fluoromethyl-ornithine
